# *Schistosoma mansoni* infection as a trigger to collapsing glomerulopathy in a patient with high-risk *APOL1* genotype

**DOI:** 10.1371/journal.pntd.0008582

**Published:** 2020-10-29

**Authors:** Precil D. Neves, Ramaiane A. Bridi, Janaína A. Ramalho, Lectícia B. Jorge, Elieser H. Watanabe, Andreia Watanabe, Luis Yu, Viktoria Woronik, Rafaela B. Pinheiro, Leonardo A. Testagrossa, Lívia B. Cavalcante, Denise M. Malheiros, Cristiane B. Dias, Luiz F. Onuchic

**Affiliations:** 1 Division of Nephrology, University of São Paulo School of Medicine, São Paulo, Brazil; 2 Division of Molecular Medicine, University of São Paulo School of Medicine, São Paulo, Brazil; 3 Division of Pathology, University of São Paulo School of Medicine, São Paulo, Brazil; London School of Hygiene and Tropical Medicine, UNITED KINGDOM

## Abstract

**Background:**

*Schistosoma mansoni* schistosomiasis (SM) remains a public health problem in Brazil. Renal involvement is classically manifested as a glomerulopathy, most often membranoproliferative glomerulonephritis or focal and segmental glomerulosclerosis. We report a case of collapsing glomerulopathy (CG) associated with SM and high-risk *APOL1* genotype (HRG).

**Case report:**

A 35-year-old male was admitted for hypertension and an eight-month history of lower-limb edema, foamy urine, and increased abdominal girth. He had a recent diagnosis of hepatosplenic SM, treated with praziquantel, without clinical improvement. Laboratory tests revealed serum creatinine 1.89mg/dL, blood urea nitrogen (BUN) 24mg/dL, albumin 1.9g/dL, cholesterol 531mg/dL, low-density lipoprotein 426mg/dL, platelets 115000/mm^3^, normal C3/C4, antinuclear antibody (ANA), rheumatoid factor (RF), and antineutrophil cytoplasmic antibodies (ANCA), negative serologies for hepatitis C virus (HCV) and human immunodeficiency virus (HIV), HBsAg negative and AntiHBc IgG positive, no hematuria or leukocyturia, 24 hour proteinuria 6.56g and negative serum and urinary immunofixation. Kidney biopsy established the diagnosis of CG. A treatment with prednisone was started without therapeutic response, progressing to end-stage kidney disease 19 months later. Molecular genetics investigation revealed an HRG.

**Conclusions:**

This is the first report of CG associated with SM in the setting of an HRG. This case highlights the two-hit model as a mechanism for CG pathogenesis, where the high-risk APOL1 genotype exerts a susceptibility role and SM infection serves as a trigger to CG.

## Author summary

Schistosomiasis mansoni is still a public health problem in Brazil and renal involvement is described. In such cases, a glomerulopathy is the typical manifestation, most often membranoproliferative glomerulonephritis. In the current article, we report a patient with a recent diagnosis of hepatosplenic SM who was admitted for nephrotic syndrome associated with reduced renal function and hypertension. Kidney biopsy established the diagnosis of collapsing glomerulopathy (CG) and molecular genetics investigation identified a high-risk *APOL1* genotype (HRG). Of note, HRG has been associated with increased risk to develop CG, and a two-hit model has been proposed for the genesis of this glomerulopathy. According to this model, a HRG represents the increased-susceptibility component, while an infection or other environmental factors could act as triggers for the development of CG. Based on those data and model, our case raises SM infection as a new trigger for this severe form of glomerulopathy. This is the first description of a case of CG associated with SM in a patient with an HRG. This case corroborates the interactive role between genetic and environmental factors in the pathogenesis of CG but also identifies SM infection as an additional trigger for its development.

## Background

*Schistosoma mansoni* schistosomiasis (SM) is caused by the helminth *Shistosoma mansoni* and is currently endemic in a few countries of South America and some countries in Africa. This parasitosis remains a public health challenge in Brazil because it is still endemic in some northeastern and central states.[1] Renal involvement is mainly expressed as glomerulopathies in SM, most often as membranoproliferative glomerulonephritis (MPGN) and focal and segmental glomerulosclerosis (FSGS). [2,3]

CG, no longer considered a histological variant of FSGS, presents a multifactorial pathogenesis, having been associated with infectious diseases, autoimmune illnesses, usage of specific medications, ischemia, mitochondrial dysfunction, and genetic factors. Notably, such factors may trigger CG in isolation or associated with each other.[4,5]

Variants in the *APOL1* gene have been associated with increased risk to develop specific kidney diseases, including FSGS and CG. [6] Interestingly, these disorders may manifest following exposure to environmental triggers, such as human immunodeficiency virus (HIV), hepatitis C virus (HCV) or parvovirus infections, or within a primary context, in which no triggering factor can be identified.[7,8]

In this report, we describe a patient with a high-risk *APOL1* genotype (HRG) who developed CG in the setting of SM, raising this helminth infection as a likely additional trigger for this severe form of glomerulopathy.

## Case report

A previously healthy 35-year-old black male was referred to our service due to new onset of hypertension and lower-limb edema, followed by progression to anasarca within an eight-month period. He had been recently diagnosed with schistosomiasis by serological test (ELISA) (immunoglobulin G: 5; normal range: less than 1.1). An upper abdomen ultrasound revealed liver with increased dimensions and a heterogeneous parenchyma due to diffuse periportal hyperechogenicity (consistent with periportal fibrosis) as well as signs of portal hypertension, including splenomegaly, increased portal vein caliber, and detection of hepatofugal portal venous flow by Doppler ultrasound. These findings were strengthened by the identification of esophageal and gastric varices at upper digestive endoscopy. The patient referred no other systemic complaints or previously diagnosed comorbidities. He reported a brother also recently diagnosed with schistosomiasis but denied family history of renal diseases. His physical examination disclosed systemic hypertension (blood pressure of 157/106mmHg), anasarca, semiology consistent with bilateral pleural effusion and ascitis, and +4/+4 edema in lower limbs.

Laboratory tests revealed serum creatinine of 1.89mg/dL (normal range [NR]: 0.7–1.2 mg/dL), estimated glomerular filtration rate (eGFR) by Chronic Kidney Disease Epidemiology Collaboration (CKD-EPI) equation 54 mL/min per 1.73 m^2^, blood urea nitrogen (BUN) of 24 mg/dL (NR: 4.5–24 mg/dL), electrolyte and acid-base parameters within the normal ranges, total serum protein 4.6 g/dL (NR: 6.6–8.7 g/dL), serum albumin 1.9 g/dL (NR: 3.4–4.8 g/dL), total cholesterol 531 mg/dL (NR: less than 200 mg/dL), low-density lipoprotein 426 mg/dL (NR: less than 100 mg/dL), high-density lipoprotein 56 mg/dL (NR: more than 40 mg/dL), triglycerides 202 mg/dL (NR: less than 150 mg/dL), serum glucose 89 mg/dL (NR: 70–99 mg/dL), glycosylated hemoglobin 5.5% (NR: 4.5%–5.6%), hemoglobin 13.8 g/dL (NR: 13–18 g/dL), leucocytes 6440 per mm^3^ (NR: 4,000–11,000 per mm^3^), platelets 115.000 per mm^3^ (NR: 140,000–450,000 per mm^3^), C3 155 mg/dL (NR: 90–180 mg/dL) and C4 38 mg/dL (NR: 10–40 mg/dL), hepatitis B surface antigen (HBsAg) negative and total hepatitis B core antibody (AntiHBc) immunoglobulin G positive, urinalysis with no leukocyturia or hematuria, and 24 hour proteinuria 6.56 g (NR: less than 150 mg per 24 hours). Autoantibody test results (antinuclear antibody, rheumatoid factor, antineutrophil cytoplasmic antibodies), serologies for HCV and HIV, and serum cryoglobulins were negative, and no monoclonal protein was detected. His chest X-ray displayed signs of congestion and bilateral pleural effusion. Echocardiogram evidenced mild pericardial effusion with no hemodynamic repercussion, with the other parameters within the normal range. Ultrasound of the kidneys and urinary tract showed kidneys with normal size and appearance, with preserved corticomedullary differentiation.

**Fig 1:**
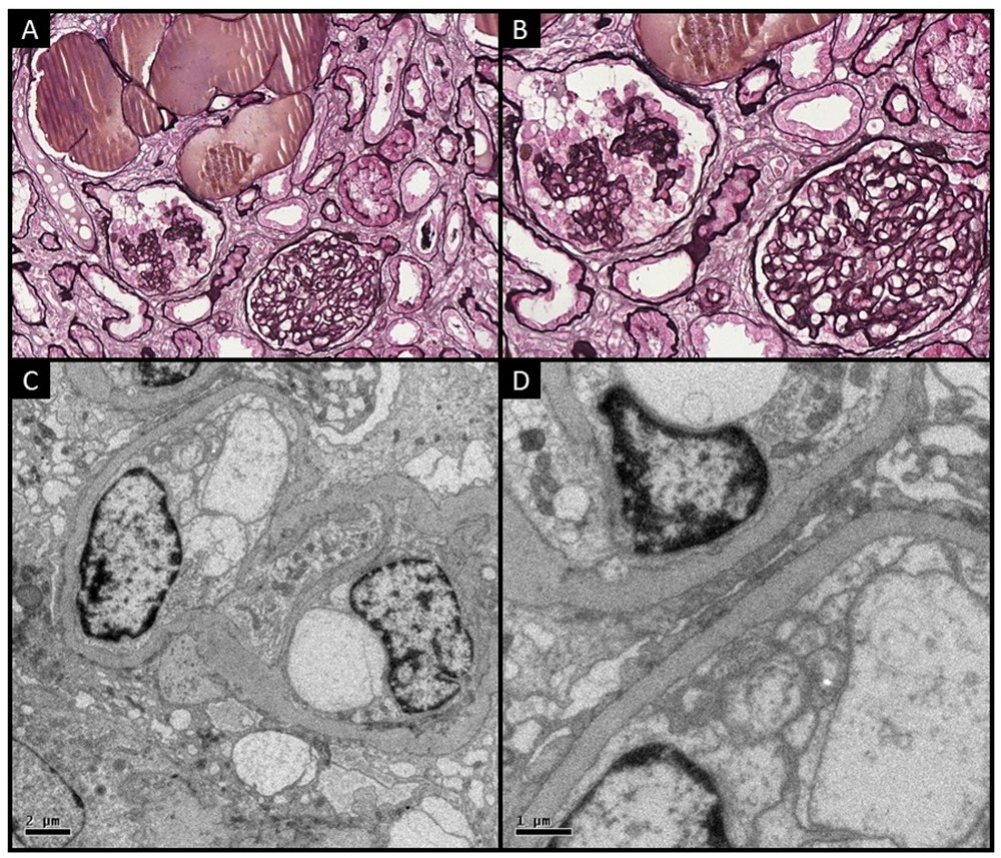
Kidney biopsy established the diagnosis of CG. (A) and (B) Light microscopy showing by Jones methenamine silver stain collapse of glomerular capillary loops (yellow arrows) and proliferation of overlying epithelial cells (green arrow), (200x and 400x, respectively); electron microscopy revealing (C) collapse of capillary walls and vacuolization of podocyte cytoplasm (6000x) and (D) effacement of podocyte foot processes (red arrows) (15000x).

In the setting of nephrotic syndrome, a kidney biopsy was performed. Light microscopy evinced 16 glomeruli, two of which were globally sclerotic, some of them with normal features and other ones with mesangial hypercellularity and capillary wall collapse, associated with marked podocyte hypertrophy and hyperplasia as well as cystic tubule dilatation (Fig 1A and 1B). Diffuse interstitial fibrosis and arteriolar hyalinosis were also observed. Immunofluorescence staining showed glomerular deposition of immunoglobulin M (+2/+3) and C3 (+3/+3) with a granular pattern and focal and segmental distribution. Electron microscopy revealed collapse of capillary walls, vacuolization of podocyte cytoplasm (Fig 1C), and global effacement of foot processes, without images consistent with viral particles, fibrillar deposits, or deposits of other nature (Fig 1D). Based on the described histological, immunofluorescence, and ultrastructural findings, the diagnosis of CG was established. Although the identification of SM antigens in the biopsy would definitely establish the cause-effect relationship between SM infection and CG, the biopsy fixation process precluded the performance of immunomicroscospy, therefore hindering this possibility.

**Fig 2:**
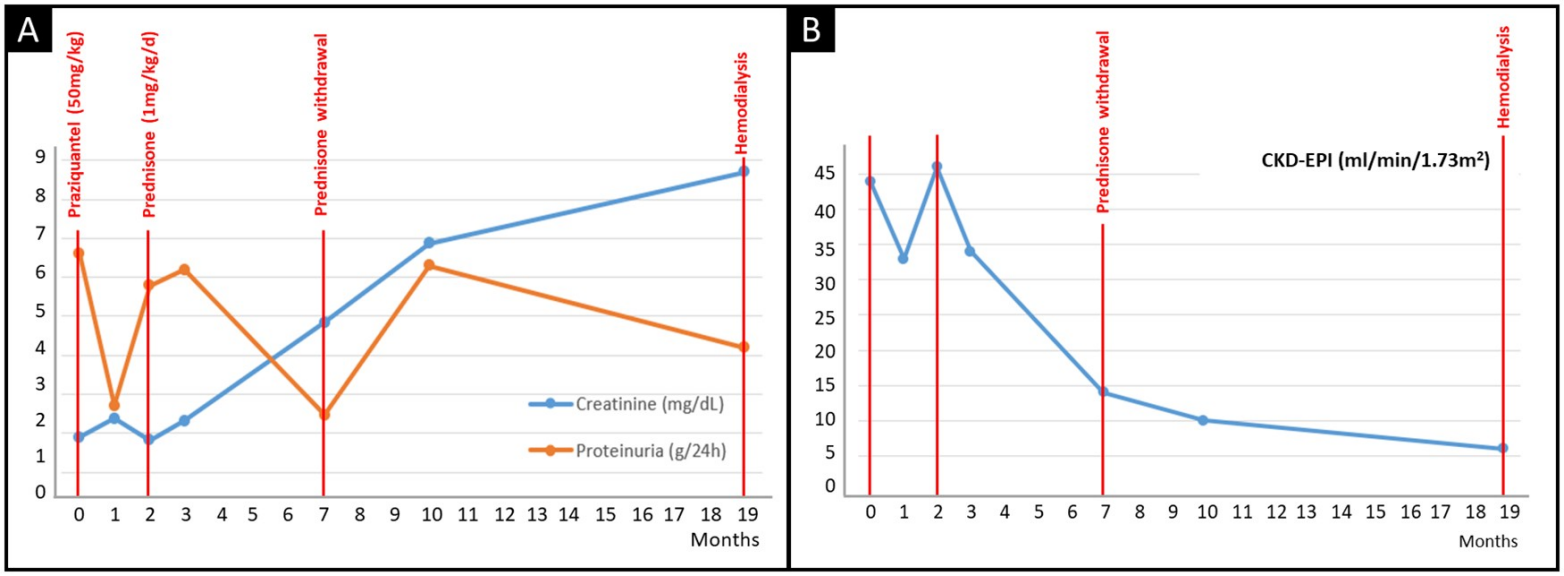
Evolutive values of (A) serum creatinine and proteinuria and (B) glomerular filtration rate measured by CKD-EPI with respective treatment from hospital admission to end-stage kidney disease.

The patient was treated with praziquantel (50 mg/kg divided into three doses and administered for one day) and enalapril (5 mg twice daily from the time of diagnosis to the start of hemodialysis), reaching partial remission of proteinuria. Such a response, however, was followed by relapse of nephrotic syndrome. In this scenario, he was started on prednisone 1 mg/kg per day for 5 months, but no therapeutic response was observed. The patient was then tested for the G1 and G2 high-risk *APOL1* alleles and found to carry a high-risk *APOL1* genotype (G1/G2). Given the lack of response to immunosuppressive therapy, he underwent a fast and progressive decrease of renal function, reaching chronic hemodialysis 19 months after the diagnosis (Fig 2).

### Discussion

*S*. *mansoni* schistosomiasis, an endemic disease in some Brazilian regions, is most often manifested by gastrointestinal symptoms and signs. Renal involvement, however, is also within the pathological spectrum of this disorder. [1,2] While glomeruli constitute the main target for kidney lesions in SM infection, glomerulopathies can be associated with the hepatosplenic or hepatointestinal forms of the disease. [3,4] The pathogenesis of such glomerulopathies, however, is still largely unknown. It has been postulated that the parasite may stimulate the immune system, leading to production of immune complexes and their subsequent deposition in glomeruli or, alternatively, it may lead to direct lesion of podocytes likely through the production of cytokines. [9]

A clinicopathological classification of SM-associated glomerulopathies was proposed by the African Society of Nephrology [10], defining five classes: class I: mesangial proliferative glomerulonephritis, representing the most frequent form; class II: exsudative glomerulonephritis; class III: MPGN; class IV: FSGS; and Class V: amyloidosis. Of note, classes III and IV are associated with progressive disease. Interestingly, in Brazilian series of patients with glomerulopathies associated with schistosomiasis, MPGN and FSGS are the most common forms. These studies also include cases of membranous nephropathy [1,2,11] not contemplated in the classification, whereas they do not report cases of amyloidosis.

A previous Brazilian study [12] analyzed the distribution of primary FSGS subtypes according to the Columbia classification. This report found a frequency of CG significantly higher than other studies worldwide, reaching a 36.6% rate, whereas in other epidemiological studies it varied from 4% to 12% [13,14]. Given the high degree of African ancestry present in the Brazilian population, epidemiological data on *APOL1* are very important to understand more broadly the kidney disease scenario in this country. Studies addressing the prevalence of HRG in Brazil and its distribution among the different regions are, however, scarce. A recent report, nevertheless, showed a 6% G1 allele frequency and 1.4% G2 allele prevalence in a healthy Brazilian population. [15] The potential impact of such frequencies upon the Brazilian chronic kidney disease (CKD) reality is, therefore, significant, although this same study revealed that *APOL1* risk variants were less frequent in dialysis patients of African ancestry in Brazil than in the United States. Remarkably, carriers of an HRG had a 10-fold higher probability to reach end-stage kidney disease (ESKD) and started dialysis substantially earlier than other CKD patients. Another Brazilian feature potentially relevant to the development of CG is the increased risk of exposure to environmental factors, particularly tropical infectious agents, which might serve as triggers to CG.

Prior experimental studies showed that podocytes containing *APOL1* risk alleles overexpress the corresponding protein when submitted to increased endogenous production of Th1 cytokines, such as interferon and Toll-like receptor agonists. This increased APOL1 protein expression, often observed in infections and autoimmune disorders, is thought to favor the development of CG. [16] Acting in line, the proposed susceptibility and triggering mechanisms support a two-hit model for the genesis of CG. Interestingly, this model has also been supported in the clinical setting, in which patients with an HRG have been reported to develop CG following viral infections, such as HIV and parvovirus, and autoimmune diseases, such as systemic lupus erythematosus. In agreement with this mechanism, transplant recipients without an HRG who received an HRG kidney developed de novo CG after cytomegalovirus or poliomavirus infection or antibody-mediated rejection. [17,18] Immune profile analysis of patients with SM revealed a role for the Th1, Th2, and Th17 lymphocytic subpopulations in the disease pathogenesis. Notably, the Th1 and Th2 profiles remain chronically activated throughout the disease process. [19,20] Taken together, the aforementioned concepts and observations strongly suggest that SM may also serve as an amplifier of mutated APOL1 protein expression in an HRG patient, triggering the development of CG.

CG is associated with a worse outcome than the histological variants that remain under the FSGS designation, even following immunosuppressive therapy and treatment of associated conditions. [14] This behavior can be appreciated in the current case, in which the patient presented partial remission after treating the parasitosis; however, nephrotic syndrome relapsed with no response to steroids and progressed to ESKD within a short time period after the diagnosis.

To our knowledge, this is the first case of CG associated with schistosomiasis mansoni in a patient with a high-risk *APOL1* genotype. This case corroborates the interactive role between genetic and environmental factors in the development of CG, highlighting the proposed two-hit model as a mechanism for its pathogenesis. Our case, therefore, not only allowed the identification of SM infection as an additional trigger for the development of CG but, also, strengthened the concept that a high-risk *APOL1* genotype exerts a susceptibility role in this glomerulopathy.

**Key learning points**

- SM is still a public health problem in Brazil;
- Renal involvement in SM is described;
- CG may be associated to *APOL1* high-risk genotype and be triggered by infectious agents.
- We report the first case of CG triggered by SM in a patient with G1/G2 high-risk genotype for *APOL1*.
